# Non-typeable *Haemophilus influenzae* major outer membrane protein P5 contributes to bacterial membrane stability, and affects the membrane protein composition crucial for interactions with the human host

**DOI:** 10.3389/fcimb.2023.1085908

**Published:** 2023-05-26

**Authors:** Yu-Ching Su, Mahendar Kadari, Megan L. Straw, Martina Janoušková, Sandra Jonsson, Oskar Thofte, Farshid Jalalvand, Erika Matuschek, Linda Sandblad, Ákos Végvári, Roman A. Zubarev, Kristian Riesbeck

**Affiliations:** ^1^ Department of Translational Medicine, Clinical Microbiology, Faculty of Medicine, Lund University, Malmö, Sweden; ^2^ European Committee on Antimicrobial Susceptibility Testing (EUCAST) Development Laboratory, c/o Clinical Microbiology, Central Hospital, Växjö, Sweden; ^3^ Department of Chemistry and The Laboratory for Molecular Infection Medicine Sweden (MIMS), Umeå Centre for Microbial Research (UCMR), Umeå University, Umeå, Sweden; ^4^ Division of Chemistry I, Department of Medical Biochemistry & Biophysics (MBB), Proteomics Biomedicum, Karolinska Institute, Stockholm, Sweden

**Keywords:** adherence, extracellular matrix, NTHI, P5, peptidoglycan, serum resistance, virulence

## Abstract

Non-typeable *Haemophilus influenzae* (NTHi) is a Gram-negative human pathogen that causes a wide range of airway diseases. NTHi has a plethora of mechanisms to colonize while evading the host immune system for the establishment of infection. We previously showed that the outer membrane protein P5 contributes to bacterial serum resistance by the recruitment of complement regulators. Here, we report a novel role of P5 in maintaining bacterial outer membrane (OM) integrity and protein composition important for NTHi-host interactions. *In silico* analysis revealed a peptidoglycan-binding motif at the periplasmic C-terminal domain (CTD) of P5. In a peptidoglycan-binding assay, the CTD of P5 (P5^CTD^) formed a complex with peptidoglycan. Protein profiling analysis revealed that deletion of CTD or the entire P5 changed the membrane protein composition of the strains NTHi 3655Δ*p5^CTD^* and NTHi 3655*Δp5*, respectively. Relative abundance of several membrane-associated virulence factors that are crucial for adherence to the airway mucosa, and serum resistance were altered. This was also supported by similar attenuated pathogenic phenotypes observed in both NTHi 3655Δ*p5* ^*CTD*^ and NTHi 3655Δ*p5*. We found (i) a decreased adherence to airway epithelial cells and fibronectin, (ii) increased complement-mediated killing, and (iii) increased sensitivity to the β-lactam antibiotics in both mutants compared to NTHi 3655 wild-type. These mutants were also more sensitive to lysis at hyperosmotic conditions and hypervesiculated compared to the parent wild-type bacteria. In conclusion, our results suggest that P5 is important for bacterial OM stability, which ultimately affects the membrane proteome and NTHi pathogenesis.

## Introduction

Non-typeable *Haemophilus influenzae* (NTHi) is a Gram-negative coccobacillus and human-restricted opportunistic pathogen (Erwin and Smith, 2007). NTHi causes a wide range of mucosal infections in the upper and lower respiratory tract. This includes acute otitis media and exacerbations in patients with asthma and chronic obstructive pulmonary disease (Leibovitz et al., 2004; Van Eldere et al., 2014; Su et al., 2018; Brown et al., 2022). Several recent reports have also suggested NTHi in causing invasive diseases (Van Eldere et al., 2014; Carrera-Salinas et al., 2021; Tonnessen et al., 2022).

NTHi possesses a plethora of mechanisms to colonize while evading the host immune system for establishment of subsequent infection. The pathogen expresses a variety of virulence factors involved in: (i) acquisition of complement regulators (*i.e.*, C4b-binding protein (C4BP), vitronectin and factor H (FH)) to suppress complement-mediated killing, and (ii) adherence to host cell receptors (*i.e.*, CEACAMs and ICAM-1) and extracellular matrix proteins (*i.e.* fibronectin, laminin, vitronectin, and collagen IV) for colonization at the airway mucosa (Hill et al., 2001; Fink et al., 2002; Avadhanula et al., 2006; Ronander et al., 2009; Klaile et al., 2013; Su et al., 2013; Langereis et al., 2014; Rosadini et al., 2014; Su et al., 2016; Su et al., 2019; Thofte et al., 2021). NTHi also occasionally harbours mutated penicillin-binding proteins (PBPs), produces β-lactamases and forms biofilm for antimicrobial resistance (Thegerstrom et al., 2018; Harrison et al., 2019; Shin et al., 2021).

**Figure 1:**
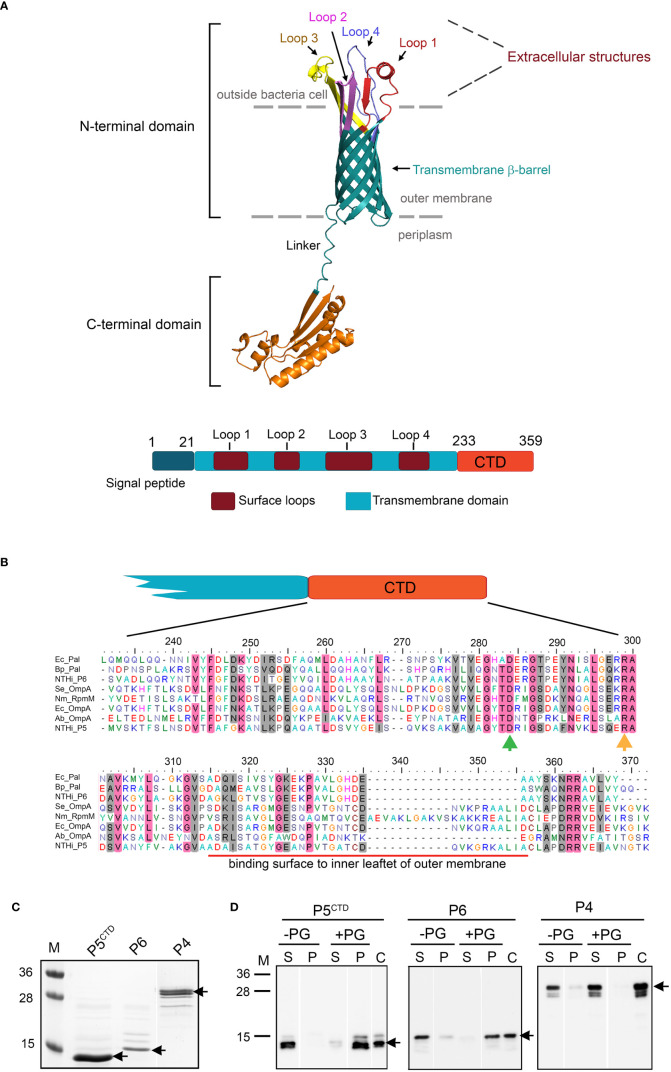
*In silico* analysis and characterization of C-terminal domain (M233-K359) of P5 (P5^CTD^). **(A)** 3D-model of P5 from NTHi 3655 based on AlphaFold prediction (Identifier: AF-A0A0H3PCS3-F1) (upper panel). The N-terminal domain of P5 forms a transmembrane β-barrel embedded within the asymmetric lipid bilayer of the outer membrane, and is connected by a linker to a C-terminal domain (CTD) sitting inside the periplasm. Four loops (Loop 1-4) form extracellular structures of P5. For clearer visualization, individual loops are indicated with different colours. The topology of P5 is similar to OmpA proteins from *E. coli* and *A. baumannii* (Park et al., 2012; Samsudin et al., 2016). Linear structural features of P5 from NTHi 3655 (lower panel). CTD of P5 is located between residue M233 and K359. **(B)** Sequence alignment of the P5^CTD^ with OmpA-like domains from other OmpA and Pal family proteins. Clustal Omega (https://www.ebi.ac.uk/Tools/msa/clustalo/ ) was used to perform multiple sequence alignment. Residue D283 (green arrow) and R298 (orange arrow) are key residues in peptidoglycan binding (Park et al., 2012). Conserved residues are in pink shading, and similar residues are in grey shading. Red line indicates the region that mediates contact with the inner leaflet of the OM. Ec_Pal, peptidoglycan-associated lipoprotein (Pal) from *Escherichia coli* (GenBank accession number: P0A912); Bp_Pal, Pal from *Burkholderia pseudomallei* (Q63RA7); NTHi_P6, P6 from NTHi 3655 (P10324); Se_OmpA, OmpA from *Salmonella typhimurium* (ACY87707.1); Nm_RpmM, OmpA from *Neisseria meningitidis* (P0A0V3); Ec_OmpA, OmpA from *E. coli* (P0A910); Ab_OmpA, OmpA from *Acinetobacter baumannii* (Q6RYW5); NTHi_P5, P5 from NTHi 3655 (EDJ92910). **(C)** Purified His-tagged P5^CTD^, P6 and P4 of NTHi 3655 on Coomassie blue-stained 12% SDS-PAGE. **(D)** Peptidoglycan-binding assay of His-tagged P5^CTD^. Of note, all His-tagged proteins were pre-treated with a peptidoglycan-removal procedure prior to the peptidoglycan-binding assay (Karalus and Murphy, 1999). Purified proteins (3 μM) incubated with 100 µg of peptidoglycan from *E. coli* K12 (+PG) were pelleted by ultracentrifugation. Supernatant (S) and pellet (P) were analysed by western blotting with HRP-conjugated anti-His pAb. Incubation without peptidoglycan (-PG) was included as a negative control. Lane C was loaded with just the His-tagged protein alone as an anti-His pAb detection control. Black arrows indicate the His-tagged proteins. M, protein marker in kDa (PageRuler™, Prestained Protein Ladder, 10-250 kDa).

One of the most studied virulence factors of NTHi is the major outer membrane (OM) protein 5 (P5). NTHi P5 is a ~35 kDa outer membrane protein (Omp) A family protein. It is composed of two main domains: (i) conserved N-terminal membrane-embedded β-barrel transmembrane domains with four highly variable and immunogenic extracellular surface loops (namely loop 1-4) and (ii) a conserved periplasmic C-terminal domain (CTD) (**Figure 1A**) (Webb and Cripps, 1998; Novotny and Bakaletz, 2003). The multifunctional surface loops of P5 contribute to NTHi adherence by binding to mucin, ICAM-1 and CEACAM1; and serum resistance by acquisition of C4BP and FH (Reddy et al., 1996; Hill et al., 2001; Avadhanula et al., 2006; Langereis et al., 2014; Rosadini et al., 2014; Thofte et al., 2021). Even though the molecular structure of P5 has been extensively investigated in the aspect of host-pathogen interactions with focus on the extracellular loops, there is a paucity of information regarding the impact of periplasmic CTD of P5 in NTHi physiology and pathogenesis. Here we report a novel role of P5 in maintaining bacterial OM integrity and membrane protein composition important for NTHi-host interactions. Our findings provide useful knowledge regarding the direct and indirect role of P5 in the pathogenesis of NTHi.

## Materials and methods

### Bacterial strains and eukaryotic cells

*Escherichia coli* DH5α (Stratagene, Santa Clara, CA) and BL21 (DE3) (Novagen, Darmstadt, Germany) were used as cloning and protein expression hosts, respectively. The genetic information and cultured conditions for *E. coli*, NTHi 3655 and the isogenic mutants are summarized in Supporting **Table S1**. Construction of *p5* ^CTD^-knockout (NTHi 3655Δ*p5^CTD^*) and *p5*-transcomplementation (NTHi 3655Δ*p5::p5*) mutants is described in Supporting **Figure S1** and **Table S2**. NTHi mutants were verified for surface display of P5 by flow cytometry using rabbit anti-peptide P5^Loop3^ polyclonal antibodies (pAbs) (Genscript, Piscataway, NJ) (Thofte et al., 2021). The human alveolar epithelial cell line A549 (ATCC CCL-185™) (American Type Culture Collection (ATCC), Manassas, VA) was maintained in F12 medium supplemented with 2 mM L-glutamine, and 10% fetal calf serum (FCS) (Gibco, Life Technologies, Carlsbad, CA).

### Isolation of membrane fractions and outer membrane vesicles

Bacterial membrane fractions and OMVs were isolated from mid and late-log phase cultures, respectively as described (Su et al., 2019; Jalalvand et al., 2022). Briefly, membrane fractions were pelleted from the supernatant of sonicated bacterial lysate at 150,000×g at 4°C for 1 hour. Bacterial lysates or membrane fractions were separated on 12% SDS-PAGE followed by western blotting. Anti-peptide P5^Loop3^ pAb and horse radish peroxidase (HRP)-conjugated donkey anti-rabbit pAb (Abcam, Cambridge, UK) were used to detect P5 on the blots (Thofte et al., 2021). The OM fractions (from three replicated preparations) were subjected to proteomic analysis (Proteomics Biomedicum, Karolinska Institutet, Sweden) (**Table S3** and **Supporting Methodology**). The yield and particle size of purified OMVs were determined by nanoparticle tracking analysis (NTA) with a NanoSight NS300 (Malvern Panalytical, Malvern, UK).

### Recombinant protein expression

DNA fragment encoding P5^CTD^ (M233 to K359) was amplified from plasmid P5^3655^-pET16b (Thofte et al., 2021) using specific primers (**Table S2**) and cloned into the expression vector pET26(b)+ (Novagen). His-tagged recombinant protein was expressed in *E. coli* BL21 (DE3) with 1 mM IPTG induction and purified as described (Su et al., 2013).

### Peptidoglycan-binding assay

The assay was performed as described (Fan et al., 2007; Park et al., 2012). Briefly, 3 µM of His-tagged recombinant protein was incubated with 100 µg of peptidoglycan from *E. coli* K12 (Invivogen, Toulouse, France) in 100 µl of ice-cold binding buffer (10 mM Tris-Cl, 10 mM MgCl_2_, 50 mM NaCl, pH6.8) at 4°C for 16 hours. Mixtures were pelleted at 350,000×g at 4°C for 1 hour. The supernatants were collected as unbound protein; the pellets were washed twice with the ice-cold binding buffer. Finally, the pellets were dissolved in 2% SDS. The supernatants and pellets were analysed on 12% SDS-PAGE and western blotting. His-tagged proteins were detected with HRP-conjugated anti-His pAb (Abcam).

### Adherence assay

Bacterial adherence to human epithelial cells was performed as described (Su et al., 2016). Briefly, cells grown to 85% confluency were infected with mid-log phase bacteria (OD_600_ = 0.5) to achieve a multiplicity of infection (MOI) of 100, at 37°C and 5% CO_2_ in F12 media without FCS for 30 min. Cells were washed with PBS and detached with trypsin-EDTA (Sigma-Aldrich, St. Louis, MO). Cell solutions were plated on chocolate agar and incubated at 37°C and 5% CO_2_ for 16 hours to quantify adherent bacteria in colony-forming units (CFU).

### Fibronectin-binding assay

The assay was performed by flow cytometry as described (Su et al., 2019). Mid-log phase (OD_600_ = 0.5) bacteria (5×10^7^ CFU) were incubated with indicated concentrations of human fibronectin (Sigma-Aldrich) in 100 µl of 1% BSA at 37°C and 5% CO_2_ for 1 hour. Samples were washed and pelleted by centrifugation. Bacteria-bound fibronectin was detected with rabbit anti-human fibronectin pAb (Dako, Glostrup, Denmark) and FITC-conjugated swine anti-rabbit pAb (Dako). Samples were analysed in a BD FACSVerse™ flow cytometer (Becton-Dickinson, Franklin Lakes, NJ).

### Serum resistance assay

The assay was performed as described (Su et al., 2013). Suspension of mid-log phase (OD_600_ = 0.5) bacteria (1.5×10^3^ CFU) in 150 µl of DVBS-BSA buffer was incubated with 5% normal human serum (NHS) for indicated time points at 37°C. Samples were plated on chocolate agar and incubated at 37°C and 5% CO_2_ for 16 hours.

### Hyperosmotic assay

The assay was performed as described (Mychack et al., 2019). Bacteria grown to mid-log phase (OD_600_ = 0.5) were pelleted, washed, and resuspended in PBS to OD_60_ = 1.0. Bacterial suspensions were 10-fold serially diluted in PBS to generate 10^4^-10^9^ CFU/ml, and 2 µl from each dilution was spotted on chocolate agar supplemented with 50 mM or 100 mM NaCl. Plates were incubated at 37°C and 5% CO_2_ for 16 hours.

### Broth microdilution

Broth microdilution (BMD) was performed in three independently repeated-experiments according to ISO 20776-1 on custom freeze-dried Sensititre plates (Thermo Scientific, Basingstoke, UK) using the EUCAST recommended broth for fastidious organisms (Mueller Hinton Fastidious (MH-F) broth) to estimate minimal inhibitory concentrations (MICs) of antibiotics. MIC endpoints after 16-20 hours of incubation at 35 ± 1°C were visually evaluated according to ISO and EUCAST recommendations.

### Statistical analyses

We used GraphPad Prism^®^ 9.0 (GraphPad Software, La Jolla, CA) for statistical analyses. One- or two-way ANOVA tests were used as indicated. Differences were considered statistically significant at *P ≤* 0.05.

## Results

### The periplasmic C-terminal domain of P5 binds peptidoglycan

Based on bioinformatic analysis, P5 of NTHi 3655 belongs to the OmpA family of proteins (InterPro entry number: IPR002368) (**Figures 1A**, **S2**). An OmpA-like domain (IPR006665) was found at the C-terminal domain (CTD) of P5 (P5^CTD^) that was located between residue M233 and K359. The P5^CTD^ shared 20.6-56.2% of sequence similarity (34.6-67.2% identity) with some peptidoglycan-associated lipoproteins (Pal) and the C-terminus of other OmpA proteins that are known to have an OmpA-like domain, and experimentally bind peptidoglycan (**Figure 1B**). While the protein sequence homology of CTDs among OmpA family proteins is generally poor, two key residues (*i.e.*, D283 and R298) that were crucial for peptidoglycan binding of OmpA in *Acinetobacter baumannii* and *E. coli* were also found on the P5^CTD^ (Park et al., 2012; Samsudin et al., 2016).

The OmpA-like domain has a peptidoglycan-binding property *via* non-covalent interactions (Hancock et al., 1994; Park et al., 2012; Samsudin et al., 2016). We therefore hypothesize that P5 also might function in an OmpA-like domain-dependent peptidoglycan binding. We observed that purified His-tagged P5^CTD^ formed a complex with peptidoglycan, which was almost comparable to His-tagged P6 (positive control) (**Figures 1C, D**). P6 is a Pal with an OmpA-like domain and is known to bind peptidoglycan in NTHi (Karalus and Murphy, 1999; Berenson et al., 2005). P6 does not have a transmembrane beta-barrel domain as seen in OmpA. The negative control, His-tagged P4 of NTHi 3655 (Kemmer et al., 2001; Su et al., 2016) did not bind peptidoglycan. Taken together, our data demonstrates that the P5^CTD^ has a peptidoglycan-binding activity as predicted by *in silico* analysis thus may play a role in the OM integrity of NTHi.

### Deletion of P5 leads to altered membrane proteome composition in NTHi

The OmpA-like domain serves as an anchor between the OM layer and the peptidoglycan. The interaction is crucial for OM-cell wall stability, and the assembly of membrane and cell wall proteins (Hancock et al., 1994; Knowles et al., 2009; Park et al., 2012; Calmettes et al., 2015; Ortiz-Suarez et al., 2016; Samsudin et al., 2016; Graham et al., 2021; Mamou et al., 2022). Since we showed that P5^CTD^ of NTHi 3655 has peptidoglycan-binding activity, we hypothesized a possible role for P5 in the distribution of NTHi periplasmic and membrane protein (membrane proteome). To this end, we generated NTHi 3655Δ*p5^CTD^* that expressed CTD-deleted P5, and a P5-transcomplemented mutant (NTHi 3655Δ*p5::p5*). In addition, NTHi 3655Δ*p5* that has a full deletion of P5 was included as a control for comparison to the deletion of P5^CTD^ (Thofte et al., 2021).

**Figure 2:**
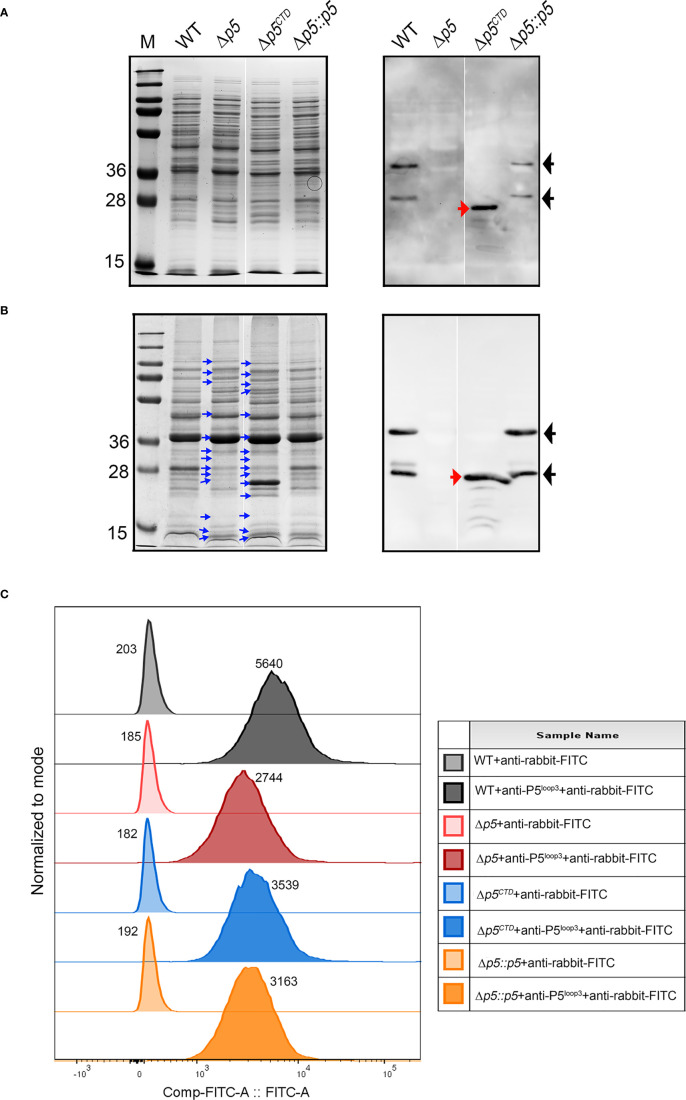
Characterization of protein profiles of NTHi 3655 wild-type and isogenic mutants. Analysis of **(A)** whole cell lysates (1×10^7^ CFU per lane) and **(B)** membrane fractions (20 µg per lane) of NTHi 3655 wild-type and isogenic mutants on a Coomassie-blue stained 12% SDS-PAGE (left panel) and western blotting (right panel). In western blotting, wild-type P5 (full length) was detected as two signal bands of ~28 kDa and 36 kDa (indicated by black arrows) from the whole cell lysate (panel A), and membrane fraction (panel B) of NTHi 3655 wild-type and NTHi 3655Δ*p5::p5*. Wild-type P5 of NTHi appears as two isoforms (~28 kDa and 36 kDa) on SDS-PAGE due to heat denaturation of protein at 85°C during sample preparation (Roier et al., 2012). In NTHi 3655Δ*p5^CTD^*, P5 with CTD deletion was detected as a signal band of ~25 kDa (indicated with red arrows) in western blotting (right panels of **A**, **B**). We did not identify any western blotting signal of P5 in the NTHi 3655Δ*p5.* Blue arrows in panel **(B)** indicate protein bands in membrane fractions that have different levels of intensities on SDS-PAGE compared to the NTHi 3655 wild-type. **(C)** Flow cytometry analysis of detection of P5 extracellular structures on the bacterial surface. Bacteria from mid-log phase (OD_600 _= 0.5) were washed and resuspended in PBS containing 1% BSA. A bacterial suspension containing 5×10^7^ CFU in 20 µl were incubated with rabbit anti-P5^Loop3^ pAb for 30 min (Thofte et al., 2021). Samples were thereafter washed followed by incubation with FITC-conjugated goat anti-rabbit IgG, and finally analyzed on a BD FACSVerse flow cytometer. For antibody background control, samples were stained with the FITC-conjugated secondary antibody only. A representative data from three independent experiments is shown. Median values of fluorescence intensity are indicated for each sample. FlowJo v10 software (BD, Williamson Way Ashland, OR) was used for data presentation. Rabbit anti-P5^Loop3^ pAb targeting extracellular loop 3 of P5 was used in western blot **(A, B)** and flow cytometry **(C)** (Thofte et al., 2021). WT, wild-type NTHi 3655; Δ*p5*, *p5*-knockout mutant (NTHi 3655Δ*p5*); Δ*p5^CTD^*, mutant expressing P5 without CTD (NTHi 3655Δ*p5^CTD^*); Δ*p5::p5, p5-*transcomplemented NTHi (NTHi 3655Δ*p5::p5*). M, protein marker (PageRuler™ Prestained Protein Ladder, 10 to 250 kDa).

Comparative SDS-PAGE of bacterial whole cell lysates and membrane fractions revealed some differences in the intensity of a few protein bands among the NTHi 3655 wild-type and mutants (**Figures 2A, B**). NTHi 3655Δ*p5^CTD^* and Δ*p5* exhibited a roughly similar pattern of protein profiling; while a simpler similarity of protein profiling pattern was observed between the wild-type and Δ*p5::p5*. In western blotting, wild-type P5 and CTD-truncated P5 were detected by anti-P5^Loop3^ pAb in NTHi 3655 and Δ*p5^CTD^*, respectively. This indicated that the deletion of CTD did not hamper protein expression of the remaining extracellular-loops and the transmembrane parts of P5 in NTHi 3655Δ*p5^CTD^*. Any corresponding signal was not detected in NTHi 3655Δ*p5* that was used as a negative control. Interestingly, we observed reduced detection of P5 on the surface of Δ*p5^CTD^* compared to the wild-type by flow cytometry analyses (**Figure 2C**). The signal was, however, higher than the P5-knockout mutant, suggesting partial or reduced exposure of P5 extracellular structures on the surface of Δ*p5^CTD^*. Unexpectedly, Δ*p5::p5* also exhibited partial detection of P5 by flow cytometry compared to the wild-type. It is currently unclear what causes the partial exposure of P5 extracellular structures in Δ*p5::p5*; it is not known if there are any spontaneous genetic mutations that could affect periplasmic post-translational modifications essential for OM insertion of P5 (Chen and Henning, 1996; Sklar et al., 2007). Since the transcomplemented strain could not fully restore the surface expression of P5, this strain was excluded from downstream experiments.

To globally understand how P5 is involved in regulating membrane protein organization, we primarily focused on analysing the proteomic composition of the membrane from Δ*p5* and compared with the wild-type strain. The bacterial membrane preparations used in this study contained mainly OM and periplasmic proteins. Based on the ranking of relative abundance, virulence factors such as major adhesins (*i.e.*, High molecular weight adhesin (HMW) and *Haemophilus* adhesin protein (Hap)) and IgA protease were less represented while P2, PD, PF and PE were more abundant in the P5-knockout mutant, with respect to the wild-type strain (**Table S3**) (Duell et al., 2016). We also noticed that periplasmic chaperones (SurA, Omp26 and DegP) and Bam complex subunit proteins (BamA, C, D and E) that are essential for proper folding and assembly of OM proteins, respectively, were less abundant in the mutant (Knowles et al., 2009; Calmettes et al., 2015; Mamou et al., 2022).

In conclusion, our results suggest that P5 has an influence on the proteomic composition of OM and periplasm in NTHi. P5 may help to maintain optimal interactions between the OM layer and the periplasmic peptidoglycan network that is crucial for cell envelope integrity and protein assembly.

### NTHi expressing CTD-deleted P5 exhibits reduced adherence to pulmonary epithelial cells and extracellular matrix protein, and is serum sensitive

The aberrant membrane proteome composition in NTHi upon the deletion of P5 is likely to affect some cellular functions that are crucial for NTHi pathogenesis (Erwin and Smith, 2007; Duell et al., 2016). Previous studies have shown that P5 deletion in different NTHi isolates results in defective adherence to host cells and increased serum sensitivity compared to the P5-expressing NTHi (Hill et al., 2001; Rosadini et al., 2014; Euba et al., 2015; Wong et al., 2016; Thofte et al., 2021).

Considering that NTHi 3655Δ*p5^CTD^* has a reduced exposure of P5 on the surface while showing almost similar membrane profiling on SDS-PAGE as the NTHi 3655Δ*p5*, we wanted to investigate whether the P5^CTD^ truncation could also affect bacterial pathogenicity in a similar fashion as when P5 was fully deleted.

**Figure 3:**
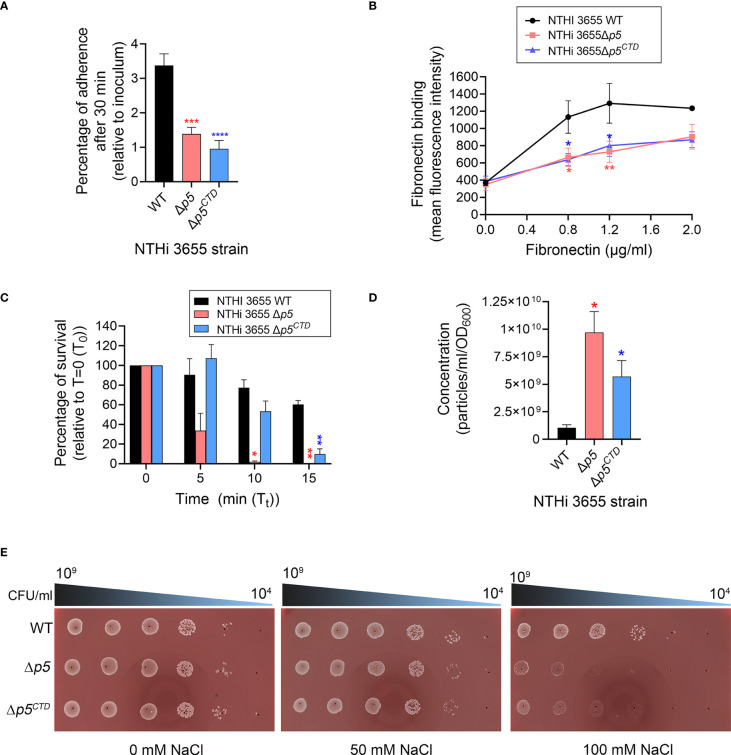
Analysis of bacterial pathogenic phenotypes and membrane stability. **(A)** Bacterial adherence to human type II alveolar epithelial cells (A549) at multiplicity of infection (MOI) of 100 for 30 min. Mean data from three independent experiments (biological replicates) is presented. Bacterial adherence was presented as percentage of CFU recovered per well relative to initial inoculum. **(B)** Binding of NTHi 3655 wild-type and mutants to human fibronectin. Bacterial (5×10^7^ CFU) binding to human fibronectin (0.8-2.0 µg/ml) in 100 µl reactions was analysed by flow cytometry after incubation for 1 hour at 37°C. Rabbit anti-human fibronectin and FITC-conjugated swine anti-rabbit pAbs were used to detect the bacterial-bound fibronectin. Data represent mean values of three independent experiments. **(C)** Serum killing of NTHi 3655 wild-type and mutants. Bacterial (1.5×10^3^ CFU) killing by 5% NHS was analysed by CFU count on chocolate agar. Heat-inactivated serum was included as a negative control and here no bacteria were killed (data not shown). Percentage of bacterial survival was expressed as (T_t_ CFU/T_0_ CFU)×100. T_0_ represents CFU of sample plated at 0 min; and Tt represents CFU of sample plated at indicated time points. Data represent mean values of three independent experiments. **(D)** Outer membrane vesicles (OMVs) production among NTHi 3655 wild-type and mutants. OMVs from bacterial cultures were sucrose-density gradient purified and subjected to nanoparticle tracking analysis with a NanoSight NS300. OMV samples were diluted in PBS until 20-120 particles per frame were archived. Settings were optimized using 100nm polystyrene beads, and samples were recorded using the same settings (camera level 12, three recordings of 30 sec each). Recordings were thereafter processed using the NanoSight 3.1 software. Data represents mean values from three independent experiments. **(E)** Spot viability assay of bacterial survival in response to hyperosmotic environment. Bacteria that were serially diluted (10^9^ to 10^4^ CFU/ml) was spotted on chocolate agar without sodium chloride (NaCl) (left panel) or supplemented with 50 mM (middle panel) and 100 mM NaCl (right panel). Images were captured using ProtoCOL 3 HD (Synbiosis, UK). The assay was repeated in three independent experiments, and images from a representative experiment were shown. For panel A-D, error bars indicate standard deviations. Differences between wild-type and mutants were calculated by one-way ANOVA for panel **(A, D)**; and two-way ANOVA for panel **(B, C)** *, *P ≤* 0.05; **, *P ≤* 0.01; ***, *P ≤* 0.005; ****, *P ≤* 0.001. WT, NTHi 3655 wild-type; Δ*p5*, *p5*-knockout mutant (NTHi 3655Δ*p5*); Δ*p5^CTD^*, mutant expressing P5 without CTD (NTHi 3655Δ*p5^CTD^*); Δ*p5::p5, p5-*transcomplemented NTHi (NTHi 3655Δ*p5::p5*).

We therefore tested the bacteria by *in vitro* functional assays related to adherence to host epithelial cells and evasion of host immune system since these two mechanisms are crucial for NTHi persistence to establish infection in the respiratory tract (Duell et al., 2016). The adherence of NTHi 3655Δ*p5^CTD^* to the lower airway A549 epithelial cells and extracellular matrix protein (fibronectin) was almost comparable to NTHi 3655Δ*p5*, which were significantly (*P ≤* 0.001) reduced compared to the NTHi 3655 wild-type (**Figures 3A, B**). When exposed to 5% NHS for 10 min, deletion of P5^CTD^ rendered the Δ*p5^CTD^* mutant to be more serum sensitive compared to the wild-type (**Figure 3C**). However, the Δ*p5^CTD^* mutant survived better (53% of survival) than the NTHi 3655Δ*p5* which was almost fully killed (1.9% survival) when exposed to NHS. The data confirm that deletion of P5^CTD^ could impair NTHi pathogenicity similarly as the full deletion of P5. We concluded that P5^CTD^ has an important impact on bacterial pathogenesis.

### NTHi 3655Δ*p5^CTD^* and Δ*p5* mutants are hypervesiculating and have compromised membrane integrity

Gram-negative bacteria with genetic knockouts of OmpA-like domain superfamily proteins (InterPro: IPR036737) are unable to establish bonds between the OM and peptidoglycan, leading to increased release of OMVs (Avila-Calderon et al., 2021). To evaluate whether the deletion of either CTD or full length P5 could affect the vesiculation of NTHi, naturally released OMVs were purified from the late-log phase bacterial culture and normalized by cell density. Both NTHi 3655Δ*p5^CTD^* and Δ*p5* produced extensive amounts of OMVs as compared to the NTHi 3655 (**Figure 3D**). Interestingly, NTHi 3655Δ*p5* was the most hypervesiculating strain, producing almost 2-fold more OMVs than the Δ*p5^CTD^* mutant (**Figure 3D**).

The increased OMV production suggested an OM instability among the Δ*p5^CTD^* and Δ*p5* mutants. To test this hypothesis, we performed an osmotic shock assay by growing bacteria on chocolate agar supplemented with different NaCl concentrations. When cultured on chocolate agar without additional NaCl or supplemented with 50 mM NaCl, similar to the wild-type, NTHi 3655Δ*p5^CTD^* and Δ*p5* mutants did not show any growth defect (**Figure 3E**). However, at a higher NaCl concentration (100 mM), NTHi 3655Δ*p5^CTD^* and Δ*p5* mutants were equally sensitive to the high salt hence showed defective growth, compared to the wild-type. In line with these results, the Δ*p5^CTD^* and Δ*p5* mutants equally displayed a 2- and 4-fold susceptibility to β-lactam antibiotics ampicillin (MIC=0.12 µg/ml) and imipenem (0.12 µg/ml), respectively compared to NTHi 3655 (MIC=ampicillin 0.25 µg/ml; imipenem 0.5 µg/ml). Collectively, our data indicate that the CTD of P5 is important in maintaining OM stability and integrity, hence bacterial vesiculation and beta-lactam antibiotics resistance.

## Discussion

**Figure 4:**
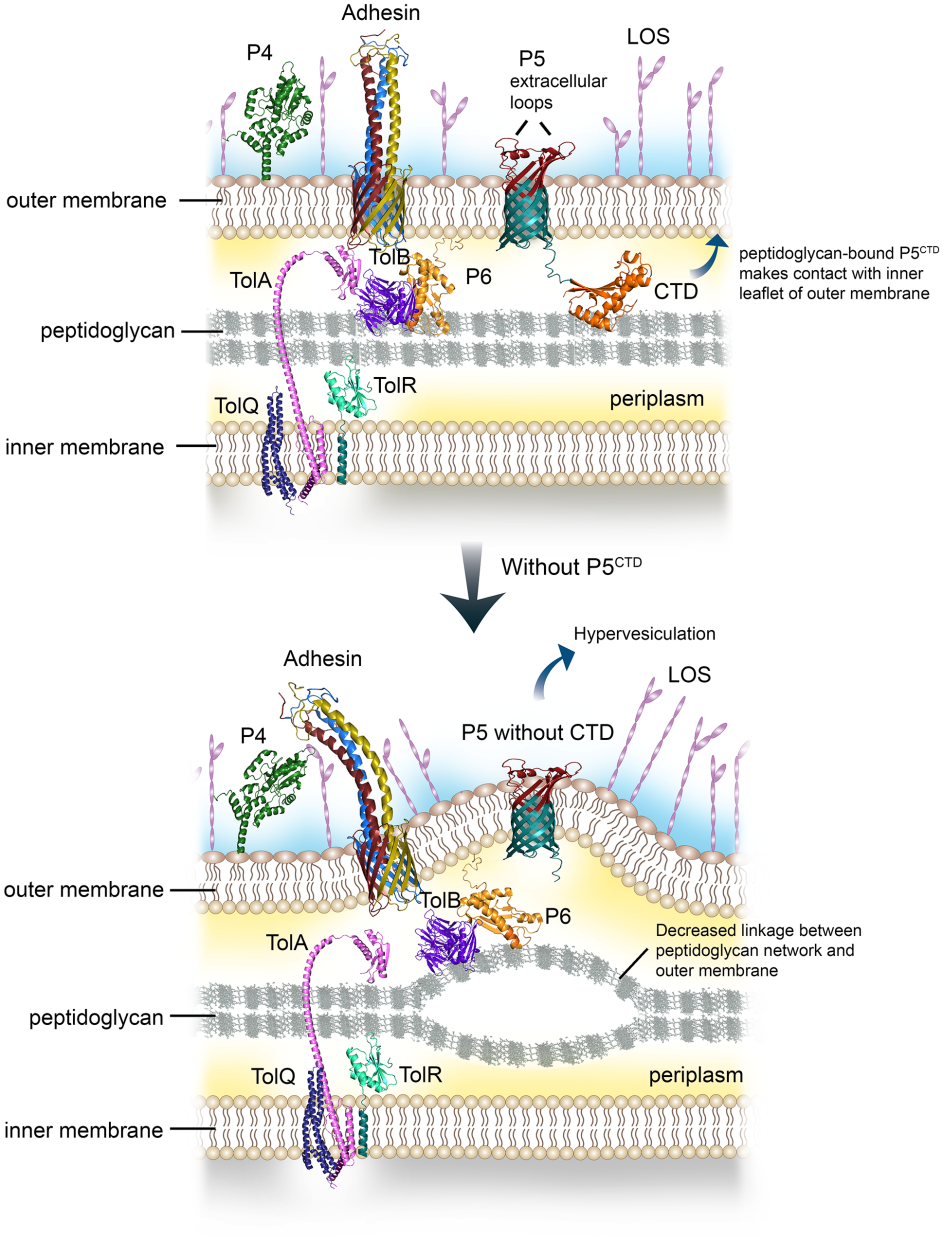
Graphical summary of the role of P5 in regulating outer membrane (OM) stability and distribution of surface virulence factors. Peptidoglycan-associated lipoprotein P6 together with P5 bind peptidoglycan *via* the OmpA-like domain (upper panel). The CTD of P5 will also interact with the inner leaflet of the OM, this will lead to stable contact between the OM and peptidoglycan. Meanwhile, TolAQRB-P6 complex joins the inner membrane with the peptidoglycan. The multiple linkages aid in the stability of NTHi cell envelope, subsequently provide an optimal membrane platform for the assembly and display of various surface virulence factors such as HMW, Hap, P4 and LOS, to name a few. However, interruption or deletion of proteins with OmpA-like domain involved in peptidoglycan binding will decrease the number of linkages or bonds between the OM and the peptidoglycan (lower panel). This will lead to OM protrusion, and increased vesiculation. The OM layer will become more permeable to solutes or antibiotics. The disrupted or collapsed OM will also hamper the translocation and assembly of various membrane proteins at bacterial surface, indirectly reducing bacterial interactions with the human host.

The OmpA-like domain has been extensively studied at the molecular level for OmpA homologs in several pathogens including *E. coli, A. baumannii, Vibrio cholerae, Neisseria meningitidis*, and *Salmonella enterica*. It was shown to contribute to cell envelope stability by forming non-covalent linkages between the OM and peptidoglycan network (Jin et al., 2011; Moon et al., 2012; Park et al., 2012; Marcoux et al., 2014; Samsudin et al., 2016). In contrast, the relevance of OmpA-like domain of P5 in NTHi cell envelope physiology hence pathogenesis has not yet been addressed. Bioinformatics analysis in combination with *in vitro* peptidoglycan-binding assays revealed the presence of the OmpA-like domain at the C-terminal of P5 that was proven to be active in forming a complex with the peptidoglycan (**Figure 1**). While the mechanism of the P5^CTD^-peptidoglycan interaction remains to be experimentally elucidated, we postulate that residues D283 and R298 of P5 might interact with the mDAP residue from the peptidoglycan. Ten residues (A313-A343) from the peptidoglycan-bound P5^CTD^ will then interact with the lipid group of the inner leaflet of OM. This in turn stabilizes the P5^CTD^ for stronger interaction with the peptidoglycan while enabling peptidoglycan to further interact with the OM. In general, P5 functions as an anchor to tether the OM to the underlying peptidoglycan, ensuring optimal periplasmic distance between the outer and inner membrane. This architecture stabilizes the OM on the NTHi cell envelope (**Figure 4**), as previously described for the OmpA-peptidoglycan models from *A. baumannii* and *E. coli* (Park et al., 2012; Samsudin et al., 2016). Our postulation is based on two rationales: (i) the annotation of D283 and R298 in P5^CTD^ that are also the key residues in *A. baumannii* OmpA for peptidoglycan binding; and (ii) the sequence similarity between the A313-A343 of P5^CTD^ (71% similarity; 74.2% identity) and the OM’s inner leaflet-binding domain of *E. coli* OmpA (**Figure S2**).

To the best of our knowledge, this is the first report in deciphering the dynamics of the P5-dependent NTHi membrane proteome. Interestingly, the aberrant protein compositions in the bacterial membrane in response to P5 deletion or truncation of P5^*CTD*^ (**Figure 2** and **Table S3**) did not alter bacterial cell morphology neither defect bacterial growth (**Figure S3**). Instead, it was more associated with a reduced pathogenic phenotype as seen in NTHi 3655Δ*p5* and to some extent, NTHi 3655Δ*p5* ^*CTD*^ (**Figure 3**). In previous reports, deletion of proteins involved in the OM and cell wall physiology (*i.e.*, VacJ, YcrB and EnvC) of NTHi also resulted in altered membrane proteome, causing the *vac*J, *ycr*B and *env*C-knockout mutants to be serum sensitive, attenuated in adherence and biofilm formation, and are hypervesiculating (Ercoli et al., 2015; Roier et al., 2016). We speculate that the reduced appearance of periplasmic chaperones (SurA, Omp26, and DegP) and BamACDE complex in the P5-knockout mutant could probably impair the maturation, and degradation of some misfolded proteins. This ultimately led to the accumulation of some OM proteins in the periplasm or the OM. This might explain the increased abundance of P2 in NTHi 3655Δ*p5*, which was probably in an unfolded/misfolded state. The impaired membrane protein assembly machineries could also affect the presentation of autotransporters (*i.e.*, Hap and HMW), or β-barrel membrane protein like CTD-deleted P5 on the surface of NTHi 3655Δ*p5* and NTHi 3655Δ*p5^CTD^*, respectively. Further experimental investigation is, however, needed to unveil the molecular mechanism on how P5 or P5^CTD^ deletion could globally alter the expression level, and protein folding state of certain membrane proteins.

The proteomic characterization of the bacterial membrane of NTHi 3655Δ*p5^CTD^* is currently ongoing, and the results will be part of a follow-up study. Therefore, besides the reduced exposure of surface P5 extracellular loops, we are currently unable to define the exact additional factors that might contribute to the NTHi 3655Δ*p5^CTD^* altered pathogenic phenotypes (**Figure 3**). We speculated that the partial exposure of P5 extracellular structures on the surface NTHi 3655Δ*p5^CTD^* could be associated with the deletion of CTD of P5 in addition to other unknown factors, suggesting that the P5^*CTD*^ might play a role in the OM insertion of P5. In *E. coli*, OM assembly of OmpA partly depends on the periplasmic post-translational modifications on the CTD, including hydrophobicity modification by oligo-(*R*)-3-hydroxybutyrates and disulphide bond formation between residues C311 and C323 (Patel et al., 2009; Qu et al., 2009; Negoda et al., 2010a ; Negoda et al., 2010b). Nevertheless, the proteomic data of NTHi 3655Δ*p5* will be used as a reference for NTHi 3655Δ*p5^CTD^* in this study, considering their almost similar protein profiling on SDS-PAGE (**Figure 2**). In addition to the absence of P5 on the bacterial surface, the decreased abundance of Hap and HMW might partly contribute to the reduced adherence of NTHi 3655Δ*p5*, and probably also for NTHi 3655Δ*p5^CTD^*, to the host epithelial cells (**Figures 2**, **3**) (Noel et al., 1994; Hill et al., 2001; Euba et al., 2015; Atack et al., 2018; Fernandez-Calvet et al., 2021). However, the reduced fibronectin binding among the mutants could be mainly attributed to the decreased density of Hap (**Figure 3**). This is because Hap has been known as the major fibronectin-binding protein in NTHi but not the P5 (Fink et al., 2002; Su et al., 2016). Moreover, the presence of P4, PF and PE did not improve the bacterial adherence, fibronectin binding, and serum resistance of NTHi 3655Δ*p5^CTD^* and Δ*p5* (Hallstrom et al., 2009; Ronander et al., 2009; Su et al., 2013; Su et al., 2016; Su et al., 2019). The reduced exposure of P5 loops on the bacterial surface of NTHi 3655Δ*p5^CTD^* might reduced the recruitment of C4BP and FH hence increased sensitivity to serum killing (**Figure 3**), as previoulsy reported for P5-knockout mutants (Langereis et al., 2014; Rosadini et al., 2014; Thofte et al., 2021).

Lipooligosaccharide (LOS) biosynthesis and assembly are related to protein assembly on the OM (Ried et al., 1990; Bulieris et al., 2003; Spahich et al., 2014). Hence, full P5 deletion might also potentially affect the assembly of LOS and the exposure of surface immunogenic antigens, however, with greater impact than the partial deletion of P5. This might cause the LOS and surface antigens of NTHi3655Δ*p5* to be more accessible to C-reactive protein or antibodies from NHS (Clark et al., 2012; Roier et al., 2012; Langereis et al., 2019), hence more complement-mediated killing of NTHi 3655Δ*p5* than the Δ*p5^CTD^*. This postulation remains, however, to be experimentally elucidated.

The loss of OM integrity was most likely caused by the absence of P5^CTD^ in both NTHi 3655Δ*p5^CTD^* and Δ*p5* mutants, leading to hypervesiculation while defective in protecting bacteria from a hyperosmotic environment (**Figures 3**, **4**) (Avila-Calderon et al., 2021; Graham et al., 2021). Despite P6 being present in Δ*p5* and probably also in Δ*p5^CTD^*, with equal abundance as in the wild-type (**Table S3**), P6 alone was not sufficient to maintain the linkage between the OM and peptidoglycan without the P5^CTD^. In *E. coli*, co-interactions among Braun’s lipoprotein, OmpA and peptidoglycan are essential in OM stability and integrity to protect bacterial cells from high internal osmotic pressure (Samsudin et al., 2016; Samsudin et al., 2017). Decreased expression of PBP has also been associated with bacterial resistance against β-lactam antibiotics (Reygaert, 2018; Kyriakidis et al., 2021). It is possible that the increased abundance of PBP3 and the compromised OM might promote killing of NTHi 3655Δ*p5^CTD^* and Δ*p5* by ampicillin and imipenem (Makover et al., 1981; Mendelman et al., 1990). A mutant of *A. baumannii* expressing OmpA without an OmpA-like domain (homolog of P5^CTD^) was also more susceptible to imipenem compared to the wild-type strain (Kwon et al., 2017).

In conclusion, our study sheds light upon the novel role of P5 which is important in regulating NTHi membrane integrity *via* the OmpA-like domain. The P5^CTD^ has an impact on the distribution of the OM proteins including a plethora of virulence factors, hence NTHi pathogenesis in general.

## Data availability statement

The original contributions presented in the study are included in the article/**Supplementary Material**. Further inquiries can be directed to the corresponding author.

## Author contributions

YCS, MK and KR coordinated the manuscript. YCS and KR drafted the manuscript. AV and RZ performed proteomic analysis. MK and FJ constructed mutants and analysed bacterial protein profiles on SDS-PAGE. MK and OT worked on OMVs preparation and analysis. MS performed bacterial fibronectin-binding assays. YCS and MK carried out bacterial adherence and hyperosmotic assays. MJ conducted surface detection of P5. SJ did bacterial serum resistance assay and growth curve study. LS analysed bacterial cell morphology on TEM. and BMD was done by EM. All authors contributed to the article and approved the submitted version.
