# Supplementary material for: Non-typeable Haemophilus influenzae major outer membrane protein P5 contributes to bacterial membrane stability, and affects the membrane protein composition crucial for interactions with the human host
Source: Front Cell Infect Microbiol. 2023 May 26;13:1085908. doi: 10.3389/fcimb.2023.1085908 (PMC10250671; doi:10.3389/fcimb.2023.1085908)
Supplement: Supplementary file 1 [file DataSheet_1.pdf]

SUPPORTING MATERIALS

Title:

Non-typeable *Haemophilus influenzae* major outer membrane protein P5 contributes to bacterial membrane stability, and affects the membrane protein composition crucial for interactions with the human host

Authors:

Yu-Ching Su[1†], Mahendar Kadari[1†], Megan L. Straw[1], Martina Janoušková [1], Sandra Jonsson[1], Oskar Thofte[1], Farshid Jalalvand[1], Erika Matuschek[2], Linda Sandblad[3], Ákos Végvári[4], Roman A. Zubarev[4], and Kristian Riesbeck[1]*

[1]Clinical Microbiology, Department of Translational Medicine, Faculty of Medicine, Lund University, Jan Waldenströms gata 59, SE-205 02 Malmö, Sweden.

[2]EUCAST Development Laboratory, c/o Clinical Microbiology, Central Hospital, SE-351 85 Växjö, Sweden.

[3]Department of Chemistry and The Laboratory for Molecular Infection Medicine Sweden (MIMS), Umeå Centre for Microbial Research (UCMR), Umeå University, 90187 Umeå, Sweden.

[4]Proteomics Biomedicum, Division of Physiological Chemistry I, Department of Medical Biochemistry & Biophysics (MBB), Karolinska Institute, Stockholm, 171 65, Sweden.

[†]These authors contributed equally.

***CORRESPONDENCE**

Kristian Riesbeck

Email: kristian.riesbeck@med.lu.se

Article type: Brief Research Report

SUPPORTING TABLES

TABLE S1: List of bacterial strains used in this study.

Bacterial strain[a, b]	Description/ genotype[c]	Reference
NTHi 3655	Otitis media clinical isolate from a 10-year-old child.	Musser et al., 1986
NTHi 3655Δ*p5*	Cm^R. Isogenic *p5* deletion mutant of NTHi 3655 by *cat* replacement. The strain is lack of P5 expression.	Thofte et al., 2021
NTHi 3655Δ$p5^{CTD}$	Cm^R. Isogenic mutant of NTHi 3655. The $p5^{CTD}$ is replaced by *cat*. The mutant expresses CTD-truncated P5.	This study
NTHi 3655Δ*p5::p5*	Zeo^R. *p5*-trans-complemented isogenic mutant of NTHi 3655Δ*p5*. The strain has a restored P5 expression as detected by SDS-PAGE and western blotting.	This study
E. coli DH5α	Used for general cloning and maintenance of plasmids.	
E. coli BL21 (DE3)	Used for protein expression upon induction with IPTG.	
E. coli-pET26b	Kan^R. *E. coli* BL21 (DE3) bearing the empty vector pET-26b.	
E. coli-P4	Kan^R. *E. coli* BL21 (DE3) bearing recombinant plasmid of pET26(b)+ with *hel* ORF (NTHi 3655) insert. The clone expresses His-tagged P4.	Su et al., 2016
E. coli-$P5^{CTD}$	Kan^R. *E. coli* BL21 (DE3) bearing recombinant plasmid of pET26(b)+ with $p5^{CTD}$ ORF (NTHi 3655) insert. The clone expresses His-tagged CTD-deleted P5.	This study
E. coli-P6	Kan^R. *E. coli* BL21 (DE3) bearing recombinant plasmid of pET26(b)+ with *p6* ORF (NTHi 3655) insert. The clone expresses His-tagged P6.	Su et al., 2019

[a] Non-typeable *Haemophilus influenzae* (NTHi) wild type and mutant strains were grown on chocolate agar or cultured in brain heart infusion (BHI) broth supplemented with NAD (Sigma-Aldrich) and hemin (Merck, Darmstadt, Germany) each at 10 μg/mL at 37°C in a humid atmosphere containing 5% CO_2.

[b] *Escherichia coli* (*E. coli*) *strains* were grown in Luria-Bertani agar or media at 37°C.

[c] Concentrations of antibiotics used: 10 µg/ml Cm; 50 µg/ml Kan; 4 µg/ml Zeo. Cm, chloramphenicol; Kan, kanamycin; Zeo, zeocin; ^{R,} Resistant.

Table S2. List of primers used.

Primer	Sequence 5’-3’	Amplicon[d]
**Construction of the *p5*^{CTD}-deletion and *p5*-transcomplemented mutant**		
KR-1_forward[a]	CGCTGTTATCCGTAGCCAGACTTAATCTATCCGAATAATTTGTTG	Fragment A: upstream region of *p5*, and *p5* gene (nt 1-696) lacking the CTD (nt 697-1080). 3’ end of the amplicon overlapped with the 5’sequence of *cat.*
KR-2_reverse	GAAGAAAAAAGTTCAATTTATTATTCAGGTGCTGCAACAACTGGTGC	
KR-3_forward	GTTGTTGCAGCACCTGAATAATAAATTGAACTTTTTTCTTCATCAG	Fragment ρ_{omp26}-*cat*: ORF of *cat.* 5’ end of the amplicon overlapped with the 3’sequence of *p5* (nt 1-696) that lack of CTD. The 3’ end of amplicon overlapped with the 5’ sequence of the downstream region of *p5*.
KR-4_reverse	GTTAAACGAATACTAAAATTACGCCCCGCCCTGCCACTCATCG	
KR-5_forward	GGCAGGGCGGGGCGTAATTTTAGTATTCGTTTAACGAAAG	Fragment B: downstream flanking region of *p5.* 5’ end of the amplicon overlapped with 3’sequence of *cat.*
KR-6_reverse[a]	CTATGGCTTGGCAGGTTTACGCATTGGTTATGCGGTATCTAATCC	
KR-7_reverse	GACCACCATTTGCCCCATATTTATTTAGTACCGTTTACCGCGATTTC	Paired with KR-1_forward to amplify the upstream region of *p5,* and *p5* gene. The amplicon is labelled as Fragment D. 3’ end of the amplicon overlapped with 5’ sequence of *ble*O.
KR-8_forward	CGGTAAACGGTACTAAATAAATATGGGGCAAATGGTGGTCACCATCC	Fragment $\rho_{bla\mathrm{TEM}}$-*ble*O: complete ORF of *ble*O. 5’ end of the amplicon overlapped

KR-9_reverse	CCAGTGATTTTTTCTCTCAGTCCTGCTCCTCGGCCACGAAGTG	with the 3' end sequence of *p5*. 3' end of the amplicon overlapped with the 5'sequence of *cat* (that lack of promoter and ATG).
KR-10_reverse	CCGAGGAGCAGGACTGAGAGAAAAAATCACTGGATATACCACCG	Paired with KR-6_reverse to amplify truncated ORF of *cat* (lack of promoter and ATG) and the downstream flanking region of *p5.* The yielded amplicon is labelled as Fragment E. 3' end of the amplicon overlapped with 3' end sequence of *ble*O.
**Cloning of full-length and P5^{CTD} fragments into pET26b**		
KR-11_forward[b]	CGGCGGATCCGATGGTAAGCAAAACTTTCAG	Complete ORF of *p5*^{CTD} (M233-K359)
KR-12_reverse[c]	CCCCAAGCTTTTTAGTACCGTTTACCGCGA	

[a] The primer pair were used to generate linear DNA as a mutation vector in knocking out *p5*^{CTD} and *p5* transcomplementation in NTHi 3655 and NTHi 3655Δ*p5*, respectively.

[b, c] Restriction enzyme cutting sites are underlined. [b]*Bam*HI, [c]*Hind*III

[d] Amplicon labelling is based on description in Figure S1.

Table S3: Label free proteomic analysis for comparison of relative abundance of membrane proteins between NTHi 3655 and NTHi 3655Δ*p5* with focus on the first 50 most abundance proteins identified in the wild type and some other important virulent factors.

Locus tag[a]	GenBank accession number	Protein description[b, c]	NTHi 3655 wild type		NTHi 3655Δ*p5*	
			Abundance[d] (grouped)	Ranking of relative abundance[c]	Abundance[d] (grouped)	Ranking of relative abundance[c]
CGSHi3655_06069	EDJ92910	**Outer membrane protein P5**	63273540915	1	n.d.	-
CGSHi3655_08469	EDJ92095	Elongation factor Tu	22560309347	2	6328607399	6
CGSHi3655_04579	EDJ92235	**Lipoprotein E (P4)**	20203504544	3	9058056604	4
CGSHi3655_05074	EDJ92441	Elongation factor G	16421155426	4	9403018225	2
CGSHi3655_06999	EDJ93618	D-galactose-binding periplasmic protein	15436745601	5	4480807308	12
CGSHi3655_05494	EDJ92795	**HMW1B, OMP-85-like protein required for secretion of HMW1A and HMW2A**	13939232618	6	4909453976	11
CGSHi3655_02399	EDJ93375	**Peptidoglycan-associated protein (P6)**	12354893925	7	6029515187	7
CGSHi3655_06394	EDJ92975	Cysteine synthase	11677252146	8	1445383519	31
CGSHi3655_07569	EDJ93732	**Outer membrane protein assembly factor BamA**	11322736675	9	4379086597	13
CGSHi3655_05044	EDJ92435	2',3'-cyclic nucleotide 2'-phosphodiesterase/3'-nucleotidase bifunctional periplasmic protein	10245205879	10	2282686411	20
CGSHi3655_07554	EDJ93729	Elongation factor Ts	10194083052	11	5326075349	9
CGSHi3655_04549	EDJ92229	**Glycerophosphoryl diester phosphodiesterase (PD)**	8796305964	12	9214407784	3
CGSHi3655_07564	EDJ93731	**Outer membrane protein 26**	8754998794	13	1024683915	50
CGSHi3655_03601	EDJ92719	**Periplasmic serine endoprotease DegP-like**	8753653475	14	375812250,7	109
CGSHi3655_05489	EDJ92794	**HMW2A, high molecular weight adhesin 2**	8737357155	15	591026186,1	76
CGSHi3655_04941	EDJ92418	**Outer membrane protein assembly factor BamD**	7153998442	16	1187515572	39

CGSHi3655_04450	EDJ92617	L-lactate dehydrogenase	7021859704	17	1789411336	25
CGSHi3655_05279	EDJ92482	Chaperonin GroEL	6685334173	18	3114618508	15
CGSHi3655_01874	EDJ93270	Uridine phosphorylase	6543015797	19	2180182518	22
CGSHi3655_01709	EDJ93237	Excinuclease ABC subunit A	6019392563	20	1560470448	27
CGSHi3655_01201	EDJ92152	MetQ/NlpA family lipoprotein	5994694377	21	6650014269	5
CGSHi3655_05369	EDJ92500	Phosphoglycerate kinase	5586139195	22	2473946498	19
CGSHi3655_05664	EDJ92829	Putative ABC-type Co2+ transport system, periplasmic component	5540286273	23	1201029652	38
CGSHi3655_06269	EDJ92950	Oligopeptide ABC transporter substrate-binding protein OppA	5319394999	24	688716870,2	66
CGSHi3655_04706	EDJ92371	High-affinity zinc uptake system protein ZnuA	4909379862	25	2793166523	16
CGSHi3655_03836	EDJ92766	Penicillin-binding protein activator LpoA	4823698873	26	1338065089	33
CGSHi3655_00570	EDJ92186	NAD nucleotidase	4704096353	27	5439999029	8
CGSHi3655_08691	EDJ92991	hypothetical protein	4145406674	28	2591290365	18
CGSHi3655_02404	EDJ93376	Tol-Pal system protein TolB	3897001650	29	1138781908	44
CGSHi3655_04816	EDJ92393	Sialic acid-binding periplasmic protein SiaP	3734750382	30	1899973049	24
CGSHi3655_02879	EDJ93471	**Putative peptidyl-prolyl cis-trans isomerase SurA**	3679725016	31	753851102,3	63
CGSHi3655_08696	EDJ92992	TolC family protein	3592711694	32	1528967870	28
CGSHi3655_07154	EDJ93649	Heme-binding lipoprotein A	3517846202	33	1694747309	26
CGSHi3655_04781	EDJ92386	**Outer membrane protein P2**	3387090222	34	67955891362	1
CGSHi3655_09651	EDJ93183	Pyruvate kinase	3381401103	35	1264870880	35
CGSHi3655_03731	EDJ92745	Dihydrolipoyl dehydrogenase ProA	3359720363	36	2277343226	21
CGSHi3655_06264	EDJ92949	Transaldolase	3025891987	37	479268613,8	88
CGSHi3655_08314	EDJ93881	Amino acid ABC transporter-binding protein	2982221337	38	1087664607	49
CGSHi3655_00986	EDJ92330	5-methyltetrahydropteroyltriglutamate--homocysteine methyltransferase MetE	2835397047	39	1116048797	46

CGSHi3655_05804	EDJ92857	TonB-dependent receptor	2736273449	40	2632016223	17
CGSHi3655_06934	EDJ93605	Phosphoenolpyruvate carboxykinase (ATP)	2708640725	41	1105107534	47
CGSHi3655_03721	EDJ92743	Pyruvate dehydrogenase E1 component	2678334158	42	1231110748	36
CGSHi3655_00871	EDJ92307	UPF0319 protein	2664344595	43	605240104,3	75
CGSHi3655_02099	EDJ93315	Aspartate--tRNA ligase	2583121050	44	1136744244	45
CGSHi3655_01714	EDJ93238	**Adhesion and penetration protein autotransporter (Hap)**	2535701286	45	382770496,2	104
CGSHi3655_08881	EDJ93029	DUF4198 domain-containing protein	2523546907	46	1012219900	52
CGSHi3655_06644	EDJ93547	2,3-bisphosphoglycerate-dependent phosphoglycerate mutase	2518737292	47	1224549133	37
CGSHi3655_08359	EDJ93890	Superoxide dismutase	2486297747	48	522943169,8	86
CGSHi3655_07834	EDJ93785	Isoleucine--tRNA ligase	2482204444	49	2060506122	23
CGSHi3655_06899	EDJ93598	DNA-directed RNA polymerase subunit alpha RpoA	2448770522	50	998807096,6	53
CGSHi3655_00690	EDJ92210	**IgA-specific metalloendopeptidase**	1947199498	60	377111850	107
CGSHi3655_01412	EDJ92275	Iron-utilization periplasmic protein (FbpA)	1907680017	61	431354774,4	94
CGSHi3655_02499	EDJ93395	Outer membrane protein P1	1823558206	63	4931102179	10
CGSHi3655_00220	EDJ91974	**HMW1A, high molecular weight adhesin 1**	1397988164	77	132175165,5	181
CGSHi3655_02309	EDJ93357	**Putative metal ABC transporter substrate-binding protein Hpf (PF)**	1390388270	78	1471424118	30
CGSHi3655_01754	EDJ93246	**Outer membrane protein assembly factor BamC homolog**	1112081052	89	208789176,3	146
CGSHi3655_07079	EDJ93634	**Outer membrane protein assembly factor BamE**	1109128392	90	380033207,3	106
CGSHi3655_08034	EDJ93825	Periplasmic chaperone PpiD	1091387249	91	175981229,2	158
CGSHi3655_00724	EDJ92122	Putative phospholipid-binding lipoprotein MlaA (VacJ lipoprotein)	764456279,3	116	544954934,5	83

CGSHi3655_09751	EDJ93203	Outer-membrane lipoprotein carrier protein LolA	734889130,3	119	872556046,1	58
CGSHi3655_03980	EDJ91973	**HMW2A, high molecular weight adhesin 2**	644855575,3	130	158420187	169
CGSHi3655_07159	EDJ93650	Putative heme iron utilization protein	532735594,6	153	408338555,3	99
CGSHi3655_04936	EDJ92417	**Surface-adhesin protein E (PE)**	446304219,8	164	267039998,1	127
CGSHi3655_01624	EDJ93220	Lipoprotein NlpI	424601766,4	170	62492455,21	245
CGSHi3655_00754	EDJ92128	Hemoglobin-haptoglobin binding protein B (HgpB)	115684374	342	107814220,6	200
CGSHi3655_06234	EDJ92943	**Peptidoglycan D,D-transpeptidase FtsI (PBP3)**	112308129,9	346	152716521	170
CGSHi3655_02684	EDJ93432	Penicillin-binding protein 1A (PBP1A)	92931801,31	375	n.d.	-
CGSHi3655_07474	EDJ93713	Multidrug resistance protein A	89552839,03	381	n.d.	-
CGSHi3655_01794	EDJ93254	Heme/hemopexin-binding protein A (HxuA)	69316855,02	425	13519913,15	383
CGSHi3655_04260	EDJ92579	Serine-type D-Ala-D-Ala carboxypeptidase (PBP5)	68766358,72	427	3009893,808	490
CGSHi3655_05499	EDJ92796	**HMW2C, putative glycosyltransferase involved in glycosylation of HMW1A and HMW2A**	61910223,84	451	n.d.	-

[a] Locus tag is based on the genomic annotation of NTHi 3655 at GenBank (NCBI Reference sequence: NZ_AAZF00000000.1).

[b] Proteins that were mentioned and discussed in the main text are highlighted in bold font.

[c] Identified proteins are ordered by their relative abundance on their first identification in NTHi 3655 wild type. -, no information.

[d] Protein abundance was calculated from the sum of all unique normalised peptide ion abundances for a specific protein on each run; data represent the grouped abundance from three replicate samples. n.d., not detected.

SUPPORTING METHODOLOGY

Sample Preparation for Proteomic Analysis

Three replicates of membrane fraction sample from NTHi wild-type and mutant, in 130 µl of 100 mM Tris-HCl, pH 8.2, with protease inhibitors were supplemented with 20 µl of 8M urea in 50 mM Tris-HCl, pH 8.2 and sonicated in a water bath for 5 min. Then 20 µl of 1% ProteaseMAX in 10% acetonitrile (AcN)/50 mM Tris-HCl buffer was added before samples were sonicated in a water bath for 5 min and using a probe (a VibraCell probe (Sonics & Materials) at 2/2 s pulse with 40% amp for 1 min. Samples were transferred to a new sample tube and protein concentrations were determined by Pierce Micro BCA assay (ThermoFisher Scientific). Equal amounts (30 µg) of proteins were taken and normalized to a buffer volume of 70 µl with 50 mM Tris-HCl. Samples were reduced with 6 µl of 100 mM dithiothreitol in 50 mM Tris-HCl and incubated at 37°C for 45 min followed by alkylation with 16 µl of 100 mM iodoaceteamide in 50 mM Tris-HCl incubated at room temperature (RT) for 30 min. Proteolytic digestion was performed with addition of 5 µl of 0.1 µg/µl sequencing grade trypsin (Promega), maintaining 1:50 ratio of enzyme:protein, incubating at 37°C overnight (16 hours). Digestion was stopped by adding 6 µl of cc. formic acid (FA) before samples were cleaned on HyperSep C-18 plate with 40 µl bed volume (Thermo Fisher Scientific) using the manufacturer's instructions. Eluted peptides were dried on a vacuum concentrator (Eppendorf).

LC-MS/MS Data Acquisition and Data Analysis

Peptides reconstituted in solvent A (2% AcN/0.1% FA) were analyzed on an Ultimate 3000 UHPLC (Thermo Fisher Scientific) hyphenated to an Orbitrap™ Lumos™ mass spectrometer (Thermo Fisher Scientific, USA). Peptides were loaded in an Acclaim PepMap trap column, 2 µm x 75 µm ID x 2 cm (ThermoFisher Scientific) and separated in an EASY-Spray™ HPLC column, 2 µm x 75 µm ID x 50 cm (Thermo Fisher Scientific) using a 90 min linear gradient. Data was acquired in data dependent acquisition (DDA) mode, isolating precursors at 120,000 mass resolution in the mass range of *m/z* 375 – 1500 in 3 s cycle time. The maximum injection time (IT) of 50 ms and dynamic exclusion of 30 s, precursors were isolated with 0.7 Th width. Tandem mass spectra were obtained using high collision energy of 28%, resolution of 15,000 and maximum IT of 22 ms.

Proteins were searched against UniProt *H. influenzae* (375177) database using the search engine Mascot Server v2.5.1 (MatrixScience) in Proteome Discoverer v2.5 software (Thermo Fisher Scientific) environment allowing maximum two missed cleavages for trypsin. Oxidation of methionine, deamidation of asparagine and glutamine were set as variable modifications, while carbamidomethylation of cysteine was used as fixed modification. The false discovery rate (FDR) was set to 1%.

SUPPORTING FIGURES

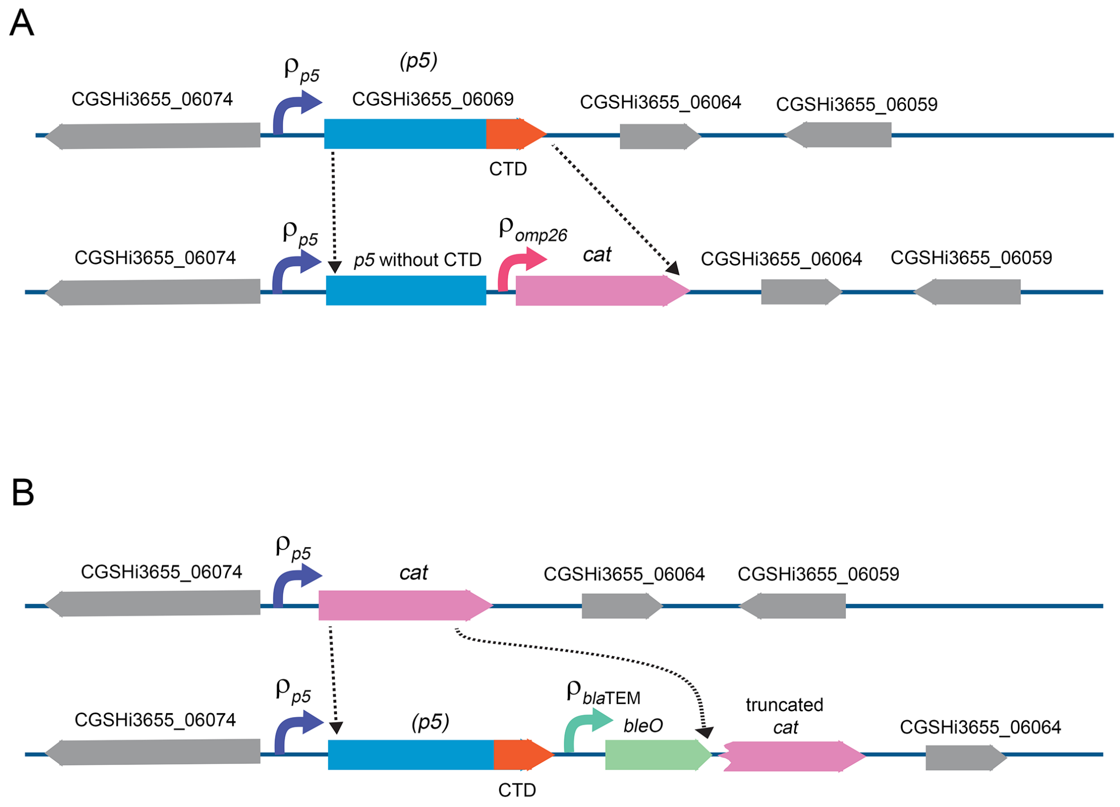

Figure S1: Schematic presentation of the construction of *p5*^{CTD}-knockout and *p5*-transcomplemented mutants in NTHi 3655. (A) *p5*^{CTD}-deletion in NTHi 3655 chromosome. Upper panel represents the loci map of *p5* (locus tag CGSHi3655_06069 (GenBank number: EDJ92910)) in NTHi 3655. *p5* gene is flanked by CGSHi3655_06074 at upstream and, CGSHi3655_06064 and CGSHi3655_06059 at downstream. Open reading frame (ORF) of CTD of P5 ($p5^{CTD}$) is located at nucleotide (nt) 697-1080 in *p5* gene. Lower panel indicates the genetic map of NTHi 3655$\Delta p5^{CTD}$. To knockout $p5^{CTD}$, the upstream (labelled as Fragment A, 1244 bp) and downstream (Fragment B, 997 bp) flanking regions of the $p5^{CTD}$ were amplified by PCR from NTHi 3655 genomic DNA using specific primer listed in Table S2. Fragment A consists of partial 5' end of CGSHi3655_06074, promoter *p5* (ρ_{p5}) and 5' part of *p5* ORF (nucleotide 1-696)). Fragment B contain CGSHi3655_06064 and partial 3' end of CGSHi3655_06059. Of note, an additional stop codon (TAA) was added at the 3' end of the partial ORF of *p5* (nucleotide 1-696+TAA) to ensure the expression of P5 lacking CTD. The ORF of antibiotic resistance genes of chloramphenicol acetyltransferase (*cat*) (AY219687.1) was amplified from our in-house designed synthetic plasmid pUC-ρ_{omp26}-Cm (GenScript) using specific primers (Table S2). Transcription of *cat* was driven by promoter (ρ_{omp26}) of *omp26* (CGSHi3655_07564) of NTHi 3655. The ρ_{omp26}-*cat* (806 bp) was then fused to Fragment B by overlapping PCR to create Fragment C (1760 bp). Overlapping PCR was performed to combine Fragment A and C and generate a linear $p5^{CTD}$-knockout vector (2958 bp). The linear DNA vector was transformed into NTHi 3655 wild type to replace the *p5*^{CTD} (nt 697-1080) with ρ_{omp26}-*cat* while retaining the ORF of the remaining transmembrane and extracellular loops of P5 (nt 1-696+stop codon), yielding NTHi 3655$\Delta p5^{CTD}$ mutant. (B) Transcomplementation of *p5* into NTHi 3655Δ*p5*. Upper panel represents the loci map of *p5*-knockout mutant. Lower panel shows the genetic map of transcomplemented-*p5* into Δ*p5* background mutant. For *p5*

transcomplementation, DNA fragment (labelled as Fragment D (1620 bp)) (consists of partial 5' end of CGSHi3655_06074, promoter *p5* (ρ_{p5}) and full ORF of *p5* (nucleotide 1-1080)) was first amplified from NTHi 3655 wild type genomic DNA with specific primers listed in Table S2. Second DNA fragment (Fragment E (1656 bp)) (consists of partial ORF of *cat*, CGSHi3655_06064 and partial 3' end of CGSHi3655_06059) was amplified from the genomic DNA of NTHi 3655Δ*p5* mutant. Of note, the ORF of *cat* in Fragment E is no longer functional because it did not have promoter and start codon. The ORF of phleomycin/zeocin-binding protein (*ble*O) (MT445781.1) was amplified from our in-house designed synthetic plasmid pUC-$\rho_{bla\mathrm{TEM}}$-Zeo (GenScript) with specific primers (Table S2). Here the promoter ($\rho_{bla\mathrm{TEM}}$) of β-lactamase TEM of NTHi 86-028NP (ABU87507.1) was used to transcribe *ble*O. The resulting PCR product of $\rho_{bla\mathrm{TEM}}$-*ble*O (716 bp) was then fused to Fragment E in the order of 5' to 3' to form Fragment F (2328 bp) by overlapping PCR. Finally, overlapping PCRs were performed to combine Fragment D and F to generate a linear *p5*-transcomplemented vector. The DNA vector was lastly transformed into NTHi 3655Δ*p5* to disrupt the *cat* ORF and replaced with the complete ORF of *p5* with it's endogenous promoter (ρ_{p5}), generating a *p5*-transcomplemented mutant, NTHi 3655Δ*p5::p5.* For panel A and B, transformation of linear vectors into NTHi 3655 wild type and Δ*p5* was carried out according to the procedure of Poje and Redfield (2003). $p5^{CTD}$-knockout and *p5*-transcomplemented mutants were selected on chocolate agar containing Cm (10 µg/mL) and Zeo (4 µg/mL), respectively. All mutants were characterized and confirmed by DNA sequencing and growth curve analysis. SDS-PAGE, western blotting and flow cytometry were also performed for detection of P5 with rabbit anti-$\mathrm{P5}^{\mathrm{loop3}}$ polyclonal antibody (pAb) (Genscript, Piscataway, NJ) (Thofte et al., 2021).

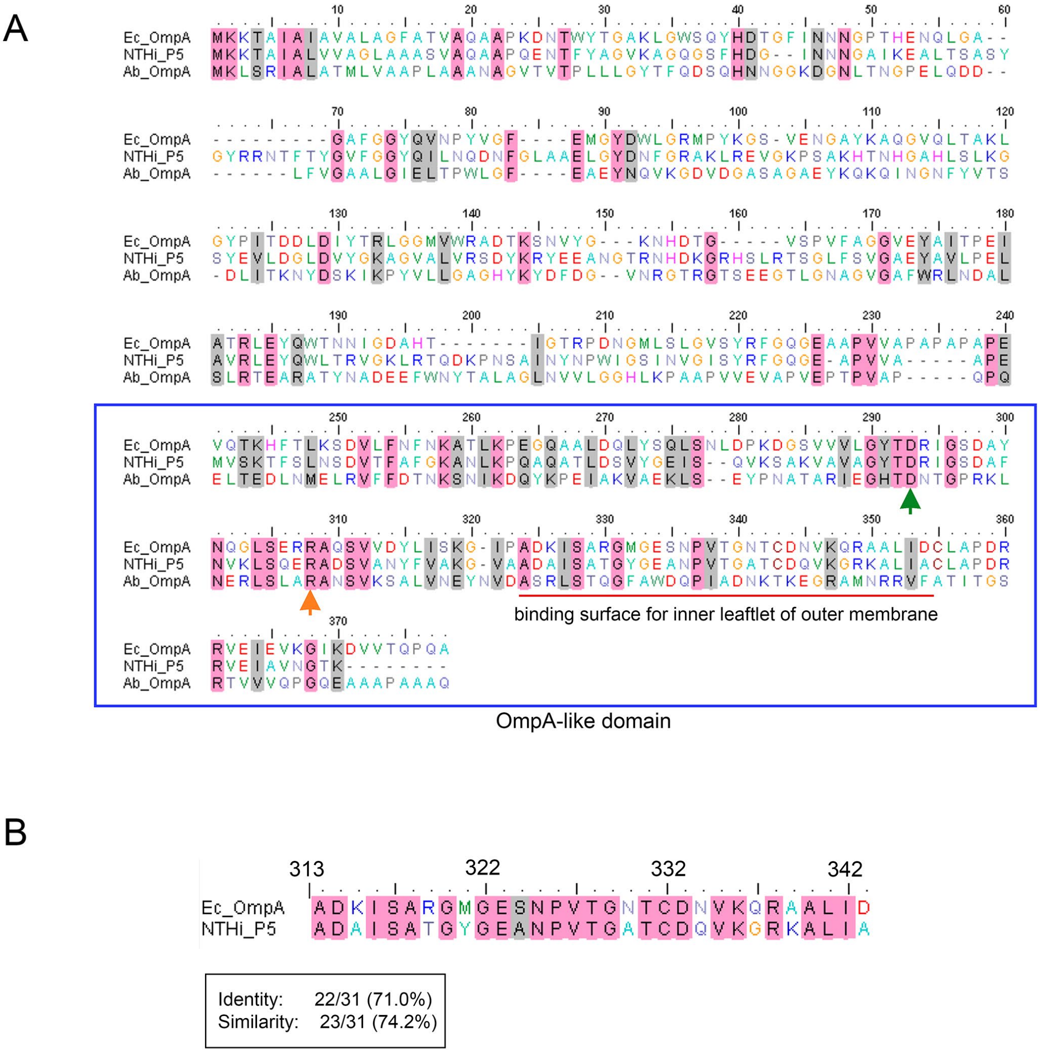

Figure S2: Sequence analysis reveals the presence of OmpA-like domain at the C-terminal (CTD) of P5. (A) Full length sequence alignment between P5 of NTHi 3655 (GenBank accession number: EDJ92910) and OmpA from *E. coli* (P0A910) and *Acinetobacter baumannii* (Q6RYW5). Clustal Omega (https://www.ebi.ac.uk/Tools/msa/clustalo/) was used to perform multiple sequence alignment. We used OmpA of *E. coli* and *A. baumannii* as the protein models for P5 since these two proteins are the most well studied OmpA-like family proteins at the molecular and structural levels (Park et al., 2012; Samsudin et al., 2016). Full length P5 of NTHi 3655 shared 46.8% and 57.9% of sequence identity and similarity with the whole molecule of OmpA from *E. coli*, respective; and 25.1% and 37.6% of sequence identity and similarity with OmpA of *A. baumannii*, respectively. Conserved residues are in pink shading, and similar residues are in grey shading, which are more apparent within the OmpA-like domain (blue box). Green and orange arrows indicate two key residues D283 and R298 (residue number based on P5 protein sequence) that are essential in peptidoglycan binding. Red line indicates region that form contact with the inner leaflet of outer membrane (OM). (B) Sequence homology of "inner leaflet of OM"-binding region (A313 and A343) between *E. coli* OmpA of and NTHi P5. Interestingly, region between A313 and A343 of P5 shared reasonable sequence similarity (74.2%) with the OM-binding surface of OmpA. This suggests the

possibility of CTD of P5 to interact with lipid group of the inner leaflet of OM as observed for *E. coli* OmpA.

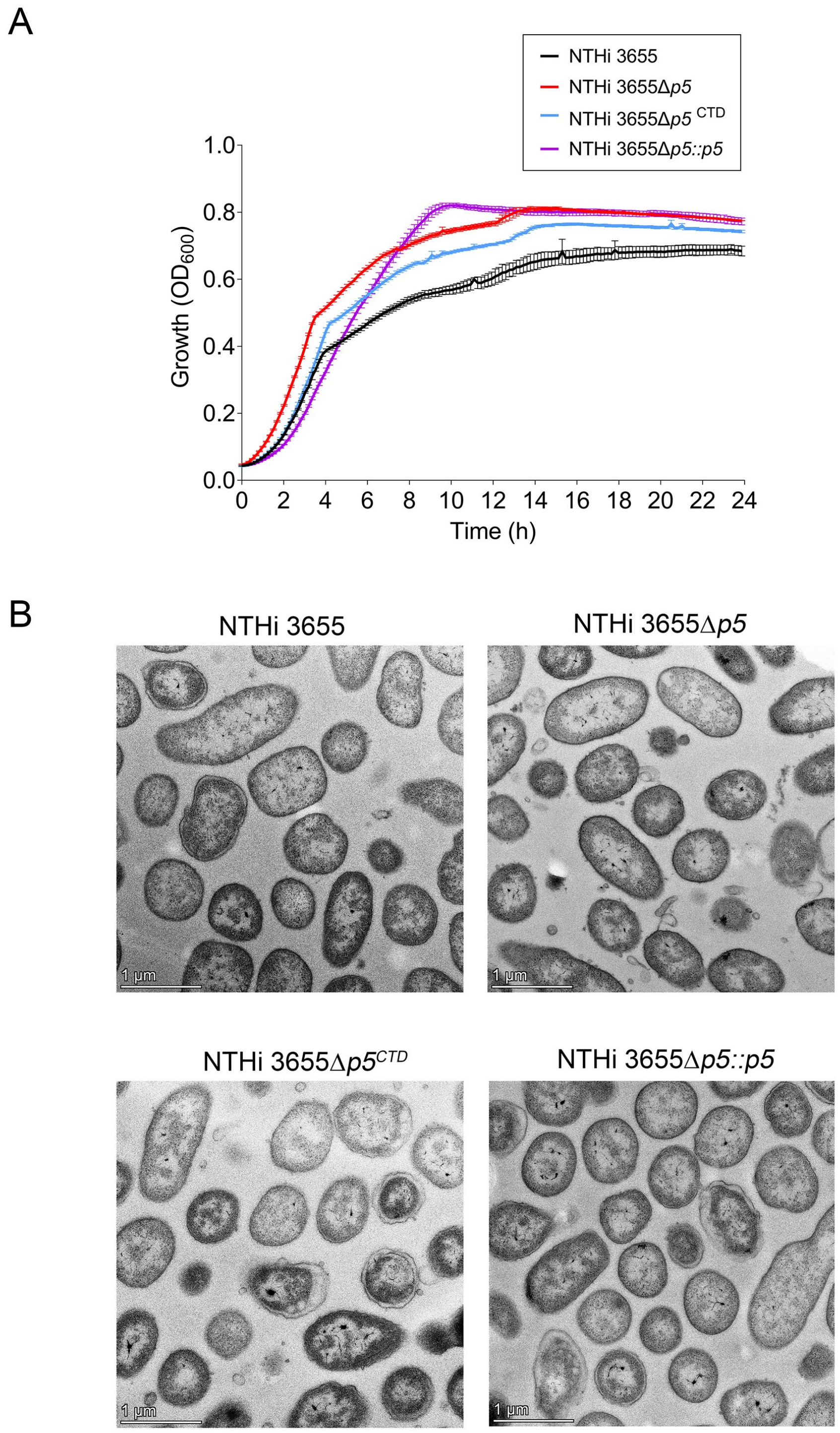

Figure S3. NTHi growth and cellular morphology in response to genetical deletion of *p5*^{CTD} or entire *p5* and *p5* transcomplentation. (A) Bacterial growth curves based on absorbance at 600 nm. Briefly, bacterial suspensions were prepared from fresh colonies and adjusted to OD_{600}=0.05 in brain heart infusion (BHI) broth supplemented with 10 µl/ml of each NAD and hemin. The bacterial suspension (100 µl) was loaded in triplicates into 96-well plates and sealed

with Breath easy seal (DiversifieD Biotech, Dedham, WA). Plate was measured using TECAN SparkControl™ multimode plate reader (Tecan, Switzerland) with the setting of 15 min interval reads for 24 hours at 37ºC, 5 % CO2, and orbital shaking (1 mm amplitude). Data were analysed using SPARK Control software Version v 3.1 SP1. Data shown is a representative experiment from three independent repeated experiments. Data represent mean values of three technical replicates derived from one representative experiment. Error bars indicate standard deviations. NTHi 3655Δ*p5* and Δ$p5^{CTD}$ mutants, as well as Δ*p5::p5* did not exhibit growth defect compared to the NTHi 3655 wild type rather a slight faster growth rate was observed among the mutants. (B) Visualization of bacterial cellular morphology by transmission electron microscope (TEM). TEM analysis were repeated three times and images from one representative analysis were shown. We did not observe any apparent changes in cell shapes and cell envelopes between the NTHi 3655 wild type, the isogenic mutants (NTHi 3655Δ*p5* and Δ$p5^{CTD}$) and the transcomplemented mutant (NTHi 3655Δ*p5::p5*). The procedure for TEM was as described in (Thofte et al., 2021). Briefly, for TEM sample preparation, bacteria colonies were picked from the plate, fixated in 2.5% glutaraldehyde phosphate buffer and treated with 1% OsO4. Samples were washed and further dehydrated in an ethanol gradient series, and then infiltrated with increasing concentrations of LR white resin in ethanol (1:3, 1:1, 3:1) and added with 100% resin. The infiltration steps were performed with PELCO BioWave (Ted Pella). Samples were polymerized overnight at 65°C, sectioned to ultrathin 70 nm ribbons using Ultracut S ultramicrotome (Leica Microsystems) with a diamond knife (Diatome), placed on formvar coated cupper grids and contrasted with uranyl acetate and lead citrate. The samples were examined with a FEI Talos L120 TEM (Thermo Fisher Scientific). Micrograph images were acquired with FEI Ceta CMOS detector (Thermo Fisher Scientific) with 16k pixels. Electron microscopy preparation and analysis were carried out at Umeå Centre for Electron Microscopy (UCEM) at Umeå university (Sweden). A scale bar indicating 1 µm is inserted into all images.

REFERENCES

Musser, J.M., Barenkamp, S.J., Granoff, D.M., and Selander, R.K. (1986). Genetic relationships of serologically nontypable and serotype b strains of *Haemophilus influenzae*. *Infect Immun* 52(1)**,** 183-191. doi: 10.1128/iai.52.1.183-191.1986.

Park, J.S., Lee, W.C., Yeo, K.J., Ryu, K.S., Kumarasiri, M., Hesek, D., et al. (2012). Mechanism of anchoring of OmpA protein to the cell wall peptidoglycan of the gram-negative bacterial outer membrane. *FASEB J* 26(1)**,** 219-228. doi: 10.1096/fj.11-188425.

Poje, G., and Redfield, R.J. (2003). Transformation of *Haemophilus influenzae*. *Methods Mol Med* 71**,** 57-70. doi: 10.1385/1-59259-321-6:57.

Samsudin, F., Ortiz-Suarez, M.L., Piggot, T.J., Bond, P.J., and Khalid, S. (2016). OmpA: A Flexible Clamp for Bacterial Cell Wall Attachment. *Structure* 24(12)**,** 2227-2235. doi: 10.1016/j.str.2016.10.009.

Su, Y.C., Mattsson, E., Singh, B., Jalalvand, F., Murphy, T.F., and Riesbeck, K. (2019). The Laminin Interactome: A Multifactorial Laminin-Binding Strategy by Nontypeable *Haemophilus influenzae* for Effective Adherence and Colonization. *J Infect Dis* 220(6)**,** 1049-1060. doi: 10.1093/infdis/jiz217.

Su, Y.C., Mukherjee, O., Singh, B., Hallgren, O., Westergren-Thorsson, G., Hood, D., et al. (2016). *Haemophilus influenzae* P4 Interacts With Extracellular Matrix Proteins Promoting Adhesion and Serum Resistance. *J Infect Dis* 213(2)**,** 314-323. doi: 10.1093/infdis/jiv374.

Thofte, O., Bettoni, S., Su, Y.C., Thegerstrom, J., Jonsson, S., Mattsson, E., et al. (2021). Nontypeable *Haemophilus influenzae* P5 Binds Human C4b-Binding Protein, Promoting Serum Resistance. *J Immunol* 207(6)**,** 1566-1577. doi: 10.4049/jimmunol.2100105.
